# Effects of Compound Yeast Culture and Yeast Cell Wall Polysaccharide on Intestinal Barrier Function in Mongolian Ram Lambs

**DOI:** 10.3390/ani16111661

**Published:** 2026-05-29

**Authors:** Songjian Li, Pengxiang Bai, Shixiong Liu, Zixuan Xu, Majigsuren Zolzaya, Dorjgoo Purevtsogt, Hui Chen, Dacheng Liu

**Affiliations:** 1 College of Veterinary Medicine, Inner Mongolia Agricultural University, Hohhot 010018, China; imaulsj@163.com (S.L.);; 2 Laboratory of Physiology and Pathology of Young Animals, Research Institute of Veterinary Medicine, Mongolian University of Life Sciences, Ulaanbaatar 17029, Mongolia

**Keywords:** compound yeast culture, Mongolian ram lambs, intestinal barrier function, gut microbiota, tight junction protein, immune barrier function

## Abstract

During the early growth stage, lambs are highly susceptible to pathogenic microbial invasion due to an underdeveloped intestinal structure, unstable microbial colonization, and immature mucosal immune function, leading to diarrhea, growth retardation, and elevated mortality factors that severely constrain the production efficiency and economic viability of the sheep industry. This study aimed to compare the regulatory effects of compound yeast culture (CYC) and yeast polysaccharides (YPs) on intestinal barrier function in Mongolian male lambs and clarify their underlying molecular mechanisms. Eighteen lambs were randomly assigned to three groups (*n* = 6/group): control group (basal diet), CYC group (40 g/kg), and YP group (3 g/kg). After a 30-day feeding trial, intestinal histomorphology, tight junction proteins, immune signaling pathways, and gut microbiota were analyzed. The results showed that both additives improved intestinal villus morphology, and CYC markedly increased the villus height/crypt depth ratio (*p* < 0.05). At the mechanical barrier level, CYC upregulated the protein expression of occludin, claudin-1, and ZO-1, whereas YPs increased occludin and ZO-1 expression (*p* < 0.05). Immunologically, CYC inhibited intestinal inflammation via the TLR4/TRAF6/MyD88/NF-κB pathway, increasing interleukin-10 (IL-10) and secretory immunoglobulin A (sIgA) while decreasing pro-inflammatory cytokines. YPs exerted similar anti-inflammatory effects through the TLR2/MyD88 pathway. Microbial analysis indicated that both additives increased the relative abundance of beneficial bacteria including *Eubacterium*, *Bacillus*, and *Succinivibrio*, while reducing the potential pathogen *Mogibacterium*. Spearman correlation analysis revealed that *Mogibacterium* was positively correlated with TNF-α and negatively correlated with occludin expression. In conclusion, CYC and YPs effectively enhance intestinal mechanical, immune, and biological barriers via different TLR-mediated pathways and microbial modulation. Both natural additives have great application potential for improving lamb health, reducing antibiotic dependence, and promoting sustainable green animal husbandry.

## Simple Summary

Compound yeast culture (CYC) is a gastrointestinal microecological preparation developed by our research team targeting the digestive tract of ruminants. Its core component is yeast polysaccharides (YPs), which consist of β-glucan (β-Glu) and mannan oligosaccharides (Man). CYC exerts beneficial effects such as promoting lamb growth, enhancing antioxidant capacity, and improving immune function. Notably, after dietary supplementation with CYC, lambs exhibited prominent phenotypic changes including intestinal mucosal thickening, villus elongation, and increased intestinal wall tension, with consistent and stable outcomes observed across multiple animal trials. Building upon these findings, the present study aims to compare the impacts of dietary supplementation with CYC and YPs on the physical, immune, and biological barrier functions of the lamb intestine, utilizing techniques including histopathology (tissue sectioning), 16S rRNA sequencing, quantitative real-time polymerase chain reaction (q-PCR), immunofluorescence, and enzyme-linked immunosorbent assay (ELISA).

## 1. Introduction

In recent years, to meet the demands of large-scale production and mitigate the ecological pressure of natural grassland grazing, the meat sheep farming model has accelerated its transition from traditional extensive grazing to intensive indoor feeding. However, this model presents challenges such as a high stocking density, frequent environmental stressors, and abrupt dietary shifts [1]. The weaning period is a critical developmental window for lambs, during which their digestive and mucosal immune systems remain immature [2]. Weaning stress often induces intestinal epithelial damage, dysbiosis, and oxidative–inflammatory imbalance, leading to high incidences of diarrhea, compromised immunity, reduced antioxidant capacity, and significantly elevated mortality [3]. These issues severely constrain growth performance and farming profitability, emerging as a core bottleneck to the high-quality development of the intensive meat sheep industry. Thus, there is an urgent need to establish a weaning stress intervention strategy centered on intestinal barrier homeostasis, with a focus on breakthroughs in key technologies including intestinal physical barrier repair, immune homeostasis reconstruction, and oxidative stress alleviation. Functional feed additives are effective tools for improving intestinal health in young ruminants and mitigating intensive farming stress [4]. Among these, microbial-derived additives such as lactic acid bacteria, yeast, and their derivatives offer advantages of high biosecurity, no drug residues, and strong targeting, enabling the synergistic regulation of intestinal microecological balance and nutrient metabolism efficiency [5]. Yeast-based additives, characterized by multiple action targets, broad adaptability, and well-documented mechanisms, have become a research focus in ruminant nutritional regulation [6]. Existing studies demonstrate that yeast cultures can reshape rumen microbial communities, enhance the apparent digestibility of roughage and concentrate, and significantly improve the growth performance of fattening sheep [7]. Dietary yeast peptides alleviate weaning-induced growth depression and intestinal damage, at least partially by modulating the jejunal microbial community structure in lambs [8]. Yeast cell wall polysaccharides (YPs), as highly purified yeast extracts, exhibit dual functions: they specifically adsorb mycotoxins, blocking their intestinal absorption and accumulation, and activate intestinal immune responses, while upregulating the expression of tight junction proteins, thereby reinforcing the physical integrity of the intestinal epithelium [9]. Their core active components, β-glucan and mannan, mediate distinct biological effects: β-glucan activates macrophages and natural killer cells via the Dectin-1 and TLR2/NF-κB signaling pathways, enhancing innate immune responses [10]; mannan competitively binds to type I fimbrial receptors of pathogens, inhibiting adhesion by *Salmonella* and *E. coli* while promoting the colonization of beneficial bacteria (e.g., Bifidobacterium, Lactobacillus), thus achieving targeted optimization of the intestinal microbiota structure.

The compound yeast culture (CYC) independently developed by our research group is prepared via standardized processes including liquid enrichment culture, solid-state anaerobic fermentation, and controlled enzymatic cell wall lysis using *Kluyveromyces marxianus* and *Saccharomyces cerevisiae* as co-fermentation strains. This formulation overcomes the limitations of single-yeast additives, containing not only highly viable yeast cells but also enriched functional metabolites such as mannan, β-glucan, small-molecule bioactive peptides, free amino acids, and short-chain organic acids. In accordance with the typical compositional standards for commercial yeast-derived additives, yeast cell wall polysaccharides (YPs) account for approximately 20~30% of the dry weight of yeast cell walls, while the yeast cell wall itself constitutes roughly 30~40% of the dry weight of intact yeast cells [11]. From this, the theoretical content of YPs in the dry weight of complete yeast cultures is estimated to be 7~10%. The YPs contained in 40 g of yeast culture are approximately equivalent to those in 3 g of purified YPs. Therefore, although the yeast culture and purified YPs were added at different mass levels in this study, they are comparable in terms of the dosage of the active component (YPs), which provides a sound dosimetric foundation for the parallel assessment of their functional effects. Preliminary pilot-scale trials showed that supplementing meat sheep diets with 0.5% CYC significantly reduced weaned lamb mortality, concurrently increasing serum IgG, IgM, and immune regulatory factors to enhance systemic immunity; it also reduced serum malondialdehyde levels and increased catalase and superoxide dismutase activities, effectively alleviating oxidative stress damage. Despite CYC’s demonstrated immune-enhancing, antioxidant, and mortality-reducing effects, its mechanism of intestinal barrier regulation remains unelucidated. Current evaluations are limited to serological and phenotypic indicators, lacking systematic analysis of key processes such as intestinal histomorphology, dynamic expression of tight junction proteins, mucosal immune microenvironment, and host microbiota interactions. This study used 2.5-month-old weaned male Mongolian lambs as a model, with three groups: control (Con), CYC, and YPs. A 30-day feeding trial was conducted, integrating multi-dimensional data—including the serum immune–inflammatory factor profiles, small intestinal histomorphology, tight junction protein expression, and detection of intestinal microbiota and intestinal immune factors—to systematically compare the synergistic regulatory effects of CYC and YPs on the mechanical, immune, and biological barriers of the lamb intestine, and to deeply analyze their distinct molecular pathways. Grounded in the realities of intensive indoor production, this study will provide a scientific basis for the precise application of CYC and YPs in weaned lambs and offer theoretical support and technical paradigms for the development of efficient, safe, and highly targeted yeast-based additives for meat sheep.

## 2. Materials and Methods

### 2.1. CYC Preparation

As detailed in our previous studies [12,13], CYC is a product of microbial fermentation generated by two highly active yeast strains, both of which are derived from the strain library of the Ruminant Microecological Preparations Research Group at the School of Veterinary Medicine of Inner Mongolia Agricultural University. For this study, fermentation was achieved through inoculation with specific activated ruminant *Kluyveromyces marxianus* (*K.marxianus*) and *Saccharomyces cerevisiae* (*S. cerevisiae*) strains at a 1:1 ratio. The raw fermentation materials consisted of 12% bran, 12% sprayed corn bran, 10% corn, 10% rice bran, 10% cotton meal, 8% wheat straw, 28% corn germ meal, and 10% soybean meal. These two yeast strains (3 × 10^8^ CFU/g each) were mixed 1:1 and used to inoculate these fermentation materials at a rate of 8% per 1000 kg, adding sterile water during stirring to a total water content of 40%. Aerobic fermentation was then performed for 72 h at 30 °C.

### 2.2. Chemical Analysis

YPs were purchased from Angel Co., Ltd. (FUBON). The dry matter (DM) contents of the CYC and YPs were measured according to AOAC (2000) method 930.15, by drying samples in a forced-air oven at 105 °C to constant weight. The N of the feed was analyzed using the Kjeldahl method of AOAC (2005) No. 984.13. The crude protein (CP) content of the feed was calculated as N × 6.25. The crude ash of feed was determined by burning the samples in a muffle furnace at 550 °C for 4 h according to the AOAC (2005) using the method No. 942.05, and the organic matter (OM) was calculated by subtracting the crude ash from DM. The organic matter (OM) (%) = total feed mass (%)—moisture content (%)—crude ash (%) (AOAC, 2005; method 942.05). Neutral detergent fiber (NDF) and acid detergent fiber (ADF) contents were determined using an automatic fiber analyzer (Ankom Technology, Macedon, NY, USA) according to the method described by Van Soest et al. [14]. respectively. The lactic acid contents were evaluated using a commercial assay kit (Nanjing Jiancheng Bio Co., Nanjing, China), according to the manufacturer’s instructions. The content of yeast β-glucan was measured using high-performance liquid chromatography in accordance with the national standard (QB/T 4572-2021). The content of yeast mannan was measured using high-performance liquid chromatography with reference to the reference group standard (T/QBAA001-2023). The nutritional and functional compositional profiles of CYC and YPs are summarized in Table 1. The CYC exhibited a dry matter content of 92.58%, crude protein concentration of 20.39%, neutral detergent fiber (NDF) content of 33.45%, and acid detergent fiber (ADF) content of 19.20%. Its bioactive polysaccharide fractions comprised β-glucan (10.5%) and mannan (9.0%), with a viable yeast cell count of 6.8 × 10^4^ CFU/g. In comparison, the YPs demonstrated significantly higher levels of crude protein (31.0%), NDF (73.0%), ADF (52.0%), β-glucan (42.0%), and mannan (20.0%), along with a dry matter content of 95.3%. Notably, the YPs contained no viable yeast cells.

### 2.3. Animals and Diet

This study obtained ethical approval for biomedical research from Inner Mongolia.

Agricultural University: [2020] 009, and was undertaken in April 2023 at a Mongolian lamb farm in Linhe District, Bayannur City, Inner Mongolia, China. The study site was located at 108°11′ E, 40°28′ N, at an altitude of 1023 m, and has a dry climate. Eighteen healthy 2.5-month-old Mongolian ram lambs (18.88 ± 0.23 kg) were selected and randomly allocated into three groups (*n* = 6/group) without stratification by body weight. Initial body weights were comparable among groups (*p* > 0.05). All lambs were individually housed in well-ventilated pens, with an activity space of 1.5 m^2^ provided for each lamb. Each pen was equipped with a feeding trough and an automatic waterer to ensure adequate access to feed and water. The control (Con) group received a standard total mixed ration (TMR) diet, while the CYC group were given a TMR diet supplemented with CYC prepared as above (40 g/kg). The lambs in the YP group received a TMR diet supplemented with YPs (3 g/kg). The feed amounts were adjusted to 2% of body weight (BW) at 10-day intervals. A 7-day pre-feeding phase was implemented, followed by a 30-day formal experiment, resulting in a total experimental duration of 37 days; deworming and inoculation occurred in the pre-trial period, and the same protocol was used to disinfect the barns for each group. The lambs received food twice a day (06:00 and 18:00) and had unrestricted access to water. The nutritional composition of the basal diet for the lambs referred to NY/T 816—2004 Feeding Standard for Meat Sheep. The basal diet formulation was identical among the three groups, while the overall dietary nutrient levels slightly differed due to the addition of different additives in the CYC and YP groups. Metabolic energy (ME) was estimated by consulting the Nutritional Value Database for Feed Ingredients and applying the energy value conversion formula for ruminant feeds. The dry matter (DM) content was determined via the 105 °C oven constant-weight method. Crude protein (CP) was quantified using the Kjeldahl nitrogen determination method. Organic matter (OM) was calculated by the ash difference method, where ash content was measured after incineration in a 550 °C muffle furnace. Neutral detergent fiber (NDF) and acid detergent fiber (ADF) were analyzed following the Van Soest detergent fiber method using a fiber analyzer. For calcium (Ca) and total phosphorus (P), samples were subjected to wet digestion prior to determination by atomic absorption spectrophotometry (AAS) and spectrophotometric colorimetry, respectively (Table 2). The feeding amounts and residual feed were measured daily, enabling the calculation of the average daily feed intake (ADFI). The lambs were weighed in the mornings before feeding on days 0 and 30 of the study, and the average daily gain (ADG) and feed-to-weight ratio (F/G) were calculated.

The specific formulas used for these calculations were as follows:

ADFI (kg/d) = [total feed amount (kg) − total residual feed (kg)]/trial days (per lamb);

ADG (kg/d) = Final body weight − Initial body weight/Total experimental days (per lamb);

F/G = ADFI/ADG (per lamb).

### 2.4. Sample Collection

After fasting overnight, all the lambs were slaughtered at 8:00 AM on day 31. Before slaughter, anesthesia with pentobarbital sodium (15 mg/kg) was induced. The jejunal contents were then removed and transferred to disposable tubes with nucleic acid protector (Bickerman Biotechnology Co., Ltd., Changde, China). After freezing in liquid nitrogen, the samples were kept at −80 °C until use. In addition, 1 cm^2^ samples of jejunal tissue were excised, incubated in 4% paraformaldehyde for H&E staining and immunofluorescence analysis, and stored at −80 °C for analyses of mRNA and protein expression in the intestinal tissues.

### 2.5. 16S rRNA Sequencing

Total DNA was extracted from the intestinal content samples of lambs in each group. The V3–V4 [15] hypervariable region of the bacterial 16S rRNA gene was amplified using the specific primer pairs (Table 3). Sequencing was undertaken on an Illumina NovaSeq instrument (Illumina Inc., San Diego, CA, USA) at Majorbio Bio-Pharm Technology Co., Ltd. (Shanghai, China), uploading the raw reads to the NCBI Sequence Read Archive (SRA) database (BioProject ID: PRJNA1183542: Gut microbiota of ram lambs). Firstly, Cutadapt (V1.9.1, http://cutadapt.readthedocs.io/en/stable/ (accessed on 20 March 2024) was employed for the quality filtering and trimming of the reads [16]. The specific criteria encompassed the following: (1) removing reads longer than 110 bases or with a variable region sequence length less than 100 bases; (2) excluding raw sequences that failed to match the barcode tag sequence; (3) deleting sequences with more than 10% of bases having a quality value lower than 20. After the aforementioned processing, the ultimate effective data (Clean Reads) were acquired [17]. The sequences were clustered into operational taxonomic units (OTUs) at 97% sequence similarity using UPARSE software (UPARSE V7.0.1001, http://www.drive5.com/uparse/ (accessed on 20 March 2024)), which is widely used and robust for gut microbiota analysis in lambs and young ruminants [18]. Next, all the OTU sequences underwent rapid multiple sequence alignment via the MUSCLE software (Version 3.8.31, http://www.drive5.com/muscle/ (accessed on 20 March 2024)) to establish their phylogenetic relationships [19]. Finally, based on the SILVA138 database (Mothur method, http://www.arb-silva.de/ (accessed on 25 March 2024)), the OTU sequences were annotated using the SSU rRNA database [20], and the community composition at each taxonomic level was analyzed.

### 2.6. qPCR

Gene-specific primers for the target immune-related genes—MyD88, TLR2, TLR4, TRAF6, IL-10, IL-12, NF-κB, TNF-α, IL-1β, IL-6, and sIgA—were designed using Primer 5 software (Premier Biosoft, Palo Alto, CA, USA) and synthesized commercially by Shanghai Sangon Biotech Co., Ltd. (Shanghai, China; sequences listed in Table 3). Total RNA was extracted from jejunal mucosal tissues using Trizol reagent (Invitrogen, Carlsbad, CA, USA) and an RNA purification kit (Tiangen Biotech Co., Ltd., Beijing, China), following the manufacturers’ protocols. The RNA concentration and purity (A260/A280 ratio) were assessed spectrophotometrically using a NanoDrop 2000 instrument (Thermo Fisher Scientific, Waltham, MA, USA). Only RNA samples with A260/A280 ratios between 1.8 and 2.0 and intact ribosomal RNA bands on 1% agarose gels were retained for downstream analysis. Complementary DNA (cDNA) was synthesized from 1 μg of qualified total RNA using a commercial reverse transcription kit (Tiangen Biotech), according to the manufacturer’s instructions. Quantitative real-time PCR (qRT-PCR) was performed on an ABI StepOnePlus Real-Time PCR System (Applied Biosystems, Thermo Fisher Scientific, Courtaboeuf, France) using SYBR Green Master Mix (Tiangen Biotech). Each reaction (20 μL final volume) contained a 100 nM concentration of each forward and reverse primer. thermal cycling conditions comprised the following: initial denaturation at 95 °C for 30 s; 40 cycles of denaturation at 95 °C for 5 s and annealing/extension at 60 °C for 30 s; a melting curve analysis (60~95 °C, increment 0.3 °C/s) to confirm amplicon specificity and the absence of primer dimers. All reactions were run in quadruplicate. β-Actin served as the endogenous reference gene for data normalization. Relative mRNA expression levels were calculated using the 2^−ΔΔCt^ method, ΔCt = Ct _target_ − Ct_β-actin_; ΔΔCt = ΔCt _treatment(CYC/YP)_ − ΔCt _control(Con)._

### 2.7. Immunohistochemistry

Tissues fixed in 4% paraformaldehyde were embedded in paraffin and cut into sections, which were then subjected to antigen repair in an appropriate buffer. Following 30 min blocking with BSA, the sections were stained overnight with antibodies specific for occludin (1:800, Proteintech, Wuhan, China), claudin-1 (1:1000), and ZO-1 (1:3000, Proteintech) at 4 °C. Sections were then rinsed with PBS, treated for 1 h at room temperature with secondary HRP goat anti-mouse IgG, re-stained, and immersed in hematoxylin for 3 min, followed by sequential immersion in 75% ethanol, 85% ethanol, and xylene for 5 min each. Sections were then sealed with neutral gum, and imaging was performed using a microscope (OLYMPUS, IXplore IX85, London, UK). Stained nuclei appeared blue, while positive DAB staining (brownish yellow) was indicative of positivity for the expression of the target tight junction proteins. The results were quantitatively analyzed using ImageJ 1.54p (National Institutes of Health, USA).

### 2.8. Immunofluorescence

After deparaffinizing paraffin-embedded sections, EDTA repair solution (pH 9.0) was used for antigen repair. After drawing a circle around each tissue sample with an H_2_O_2_ blocking pen, 5% H_2_O_2_ was added to the sections for 25 min at room temperature while they were protected from light to disrupt endogenous peroxidase activity. Slides were then rinsed three times with PBS (pH 7.4), 5 min per rinse, shaken to dry, and incubated for 30 min with 10% rabbit serum. Sections were then stained with anti-CD4 (1:1000) at 4 °C overnight, followed by the addition of appropriate HRP-conjugated goat anti-mouse antibodies. Slides were then rinsed three times with PBS (pH 7.4), 5 min per rinse, and dried slightly, and the 570 TSA dye was added to the dye circles, followed by incubation for 10 min away from light. The slides were then rinsed thrice with TBST (5 min/wash). These same steps were repeated with anti-CD8 antibodies and the 520 TSA dye. The specific fluorescence intensity of the samples was observed using a 3DHISTECH scanner (Pannoramic SCAN II, Budapest, Hungary) at excitation wavelengths of 350 nm/420 nm (DAPI), 490 nm/520 nm (520TSA), and 550 nm/570 nm (570TSA) to analyze the content of CD4 and CD8.

### 2.9. Western Immunoblotting

Tissues were ground with a tissue homogenizer, and proteins were isolated using a kit (Sangon Biotech Co., Ltd., Shanghai, China), after which a BCA kit (Sangon Biotech) was utilized to quantify the protein concentrations. Then, 50 μg of protein per sample was separated via 15% SDS-PAGE and transferred to PVDF membranes (Sangon Biotech Co., Ltd., Shanghai, China). The blots were blocked for 1 h with 5% nonfat milk, followed by treatment overnight with polyclonal antibodies specific for occludin (1:500) (Proteintech; Wuhan, China), ZO-1 (1:1000) (Proteintech), and claudin-1 (1:1000) (Proteintech) at 4 °C, and then treated for 1 h with goat anti-mouse IgG (1:10,000) (Thermo Scientific). An enhanced chemiluminescence solution (ECL, Biyuntian, Shanghai, China) was then used to detect bands with an Odyssey Infrared Imaging System (LI-COR, Bad Homburg, Germany) at 800 nm. The results were analyzed with ImageJ, normalizing ZO-1, claudin-1, and occludin levels to those of β-actin. The results were quantitatively analyzed using ImageJ (National Institutes of Health).

### 2.10. Statistical Analyses

The normality and homogeneity of variance of all data were first tested using the Shapiro–Wilk test. One-way ANOVA was performed, and SPSS 23 (SPSS Inc., Chicago, IL, USA) was used to identify differences in intestinal villus development, tight junction protein expression, and transcriptional levels and production of intestinal inflammatory factors in ram lambs. Bar charts were created using GraphPad 8.0 software. The alpha diversity (Shannon and Chao1 indices) among the groups was assessed using the Kruskal–Wallis test and post hoc Dunn Kruskal–Wallis multiple comparison with Bonferroni correction. Beta diversity based on Bray–Curtis distances was assessed by analysis of similarity (ANOSIM). The diversity analysis outputs were visualized using R (version 3.3.1).

In this study, the non-parametric Kruskal–Wallis test was used to compare relative abundance data at the genus level between groups, and the Benjamini–Hochberg method was used to correct the false discovery rate (FDR) for the results of the multiple tests. To improve analytical reliability, low-abundance genera were filtered out: only genera with relative abundance > 0.1% in at least 20% of samples were retained for downstream analysis [21,22]. This step reduces noise from sequencing artifacts and contaminant sequences. For the selected significant difference bacteria (FDR < 0.1), Dunn’s test was further used for pairwise comparison between the groups, and the trend of difference between the groups was shown by boxplots. The above analysis was completed in R (version 3.3.1), and ggpubr, FSA, rstatix and other packages were used for visualization and statistics. Because the present study used a completely randomized design with a limited sample size (*n* = 6 per group), all data were analyzed by one-way ANOVA rather than a mixed-effects model.

This study was based on Spearman correlation analysis, integrating the data of tight junction proteins, immune markers, and significantly different flora to construct a multi-omics association network. First, after removing the missing values, the pairwise Spearman correlations between the three types of variables were calculated respectively, and FDR correction was performed by the Benjamini–Hochberg method. The pairs of variables whose absolute values of correlation coefficients were greater than 0.2 and *p*-values less than 0.1 after correction were selected for constructing the network. The node layout was manually set according to the variable type, the color and transparency of the edge indicated the direction and significance of the correlation, and the size of the node reflected its network centrality, which was finally visualized through the graph package.

## 3. Results

### 3.1. CYC and YPs Influence the Growth Performance of Lambs

As presented in Table 4, initial body weight did not differ significantly among the control (Con), CYC, and YP groups (*p* > 0.05). Following a 30-day feeding period, dietary supplementation with CYC or YPs resulted in a numerical increase in final body weight relative to the Con group; however, this difference remained statistically non-significant (*p* > 0.05). In contrast, the average daily gain (ADG) over days 0~30 was significantly enhanced by both CYC and YP supplementation (*p* = 0.033), with the ADG values following the rank order CYC > YP > Con (*p* < 0.05). Initial feed intakes were comparable across all treatment groups (*p* > 0.05). By day 30, the feed intake in both the CYC and YP groups was significantly greater than that in the Con group (*p* < 0.05); however, the average daily feed intake (ADFI) over the entire 30-day period did not differ significantly among groups (*p* > 0.05). Furthermore, supplementation with CYC or YPs significantly reduced the feed-to-gain ratio (F/G) compared with the Con group (*p* < 0.05).

### 3.2. CYC and YPs Influence Jejunal Tissue Morphology in Lambs

**Figure 1:**
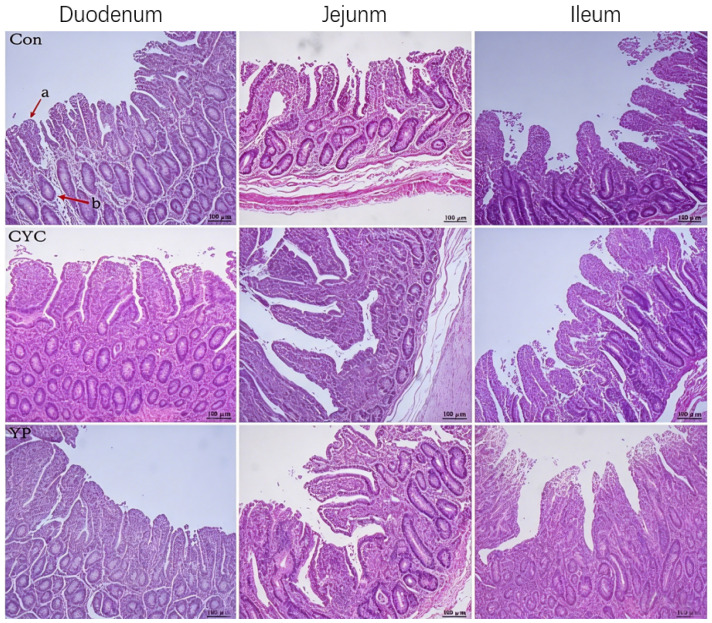
The impact of CYC and YPs on intestinal morphology and structural characteristics in lambs. a = intestinal villi; b = intestinal glands. Con = basal diet; CYC = compound yeast culture; YP = yeast cell wall polysaccharide.

When hematoxylin and eosin (H&E) staining was used to evaluate intestinal development in these lambs, microscopic analyses revealed short, thick characteristics in the intestinal villi (Figure 1). Relative to lambs in the Con group, those in the CYC and YP groups exhibited increases in villous height and crypt depth in the duodenum. A significantly greater ileal crypt depth was noted in lambs from the YP group relative to the CYC and Con groups (*p* < 0.05). The villous height to crypt depth ratio (VCR) for the jejunum and ileum was significantly greater in CYC lambs relative to those in the YP and Con groups (*p* < 0.05). Based on these results, dietary CYC or YP supplementation can promote intestinal tract development in lambs (Table 5).

### 3.3. CYC and YPs Modulate Jejunal Tight Junction Protein Expression in Lambs

**Figure 2:**
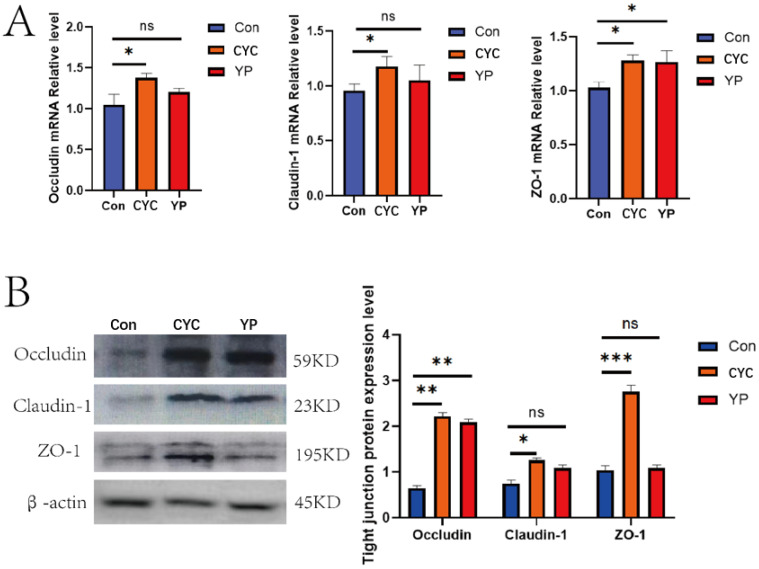
(**A**) qPCR analyses of occludin, claudin-1, and ZO-1 mRNA expression. (**B**) Western blotting analysis of occludin, claudin-1, and ZO-1 protein levels. Con = basal diet; CYC = compound yeast culture; YP = yeast cell wall polysaccharide; *n* = 6/group; * *p* < 0.05, ** *p* < 0.01, *** *p* < 0.001, ns = no significant difference.

**Figure 3:**
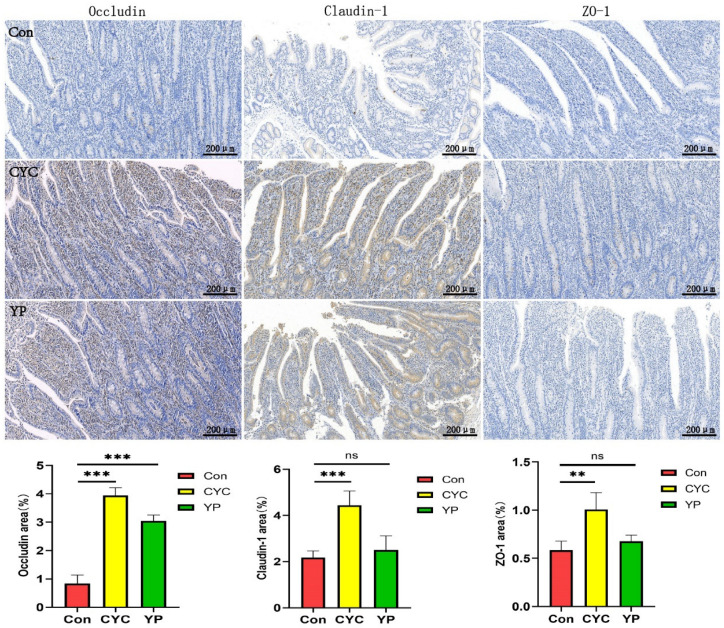
Immunohistochemical analyses of occludin, claudin-1, and ZO-1 expression. Con = basal diet; CYC = compound yeast culture; YP = yeast cell wall polysaccharide; *n* = 6/group; ** *p* < 0.01, *** *p* < 0.001, ns = no significant difference.

In qPCR analyses comparing lambs in these three experimental groups, occludin, claudin-1, and ZO-1 mRNA levels were markedly increased in the jejunal samples from the CYC group relative to the YP and Con groups (*p* < 0.05). YPs also markedly increased jejunal ZO-1 mRNA levels in these lambs relative to those in Con animals (*p* < 0.05) (Figure 2A). Western immunoblotting similarly confirmed significant increases in occludin, claudin-1, and ZO-1 protein levels in the jejunum of lambs in the CYC group, while both CYC and YPs increased occludin protein levels relative to the Con group (*p* < 0.05) (Figure 2B). Immunohistochemical staining yielded similar results in terms of the CYC-induced upregulation of occludin, claudin-1, and ZO-1 in the jejunum (*p* < 0.05). Specifically, relative to Con lambs, those in the CYC and YP groups exhibited significantly increased occludin expression (Figure 3). These results suggest that CYC more strongly promotes intestinal tight junction protein expression, with dietary CYC or YP supplementation providing an effective means of upregulating these tight junction proteins in the intestines.

### 3.4. CYC and YPs Modulate Immune-Related Gene Expression in the Jejunum of Lambs

The relative transcription levels of intestinal immune factor genes in meat sheep across different groups were assessed via quantitative polymerase chain reaction (qPCR). 

**Figure 4:**
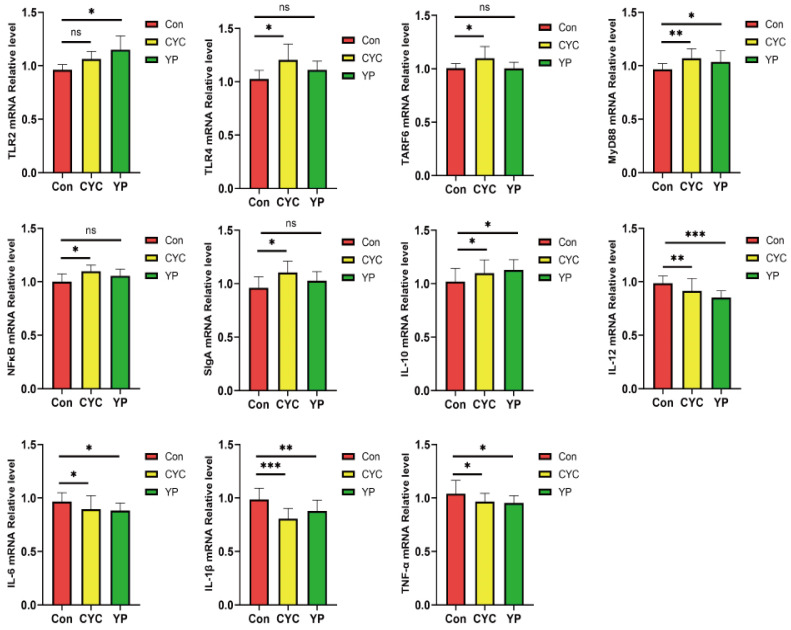
The effects of dietary CYC or YP supplementation on the mRNA levels of immune-related factors in the intestines of lambs. qPCR was used to measure TLR2, TLR4, TRAF6, MyD88, NF-κB, SIgA, IL-10, IL-12, IL-6, IL-1β, and TNF-α. TLR2 = Toll-like receptor 2; TLR4 = Toll-like receptor 4; TRAF6 = recombinant TNF receptor associated factor 6; MyD88 = myeloid differentiation primary response gene 88; NF-κB = nuclear factor kappa-B; SigA = secretory immunoglobulin A; IL-10 = interleukin-10; IL-12 = interleukin-12; IL-6 = interleukin-6; IL-1β = interleukin-1β; TNF-α = tumor necrosis factor-α. Con = basal diet; CYC = compound yeast culture; YP = yeast cell wall polysaccharide; *n* = 6/group; * *p* < 0.05, ** *p* < 0.01, *** *p* < 0.001, ns = no significant difference.

Compared with the control (Con) group, the CYC group exhibited significantly elevated relative transcription levels of the TLR4, TRAF6, MyD88, NFκB, SIgA, and IL-10 genes in intestinal tissues (*p* < 0.05). In the YP group, the transcription levels of the TLR2, MyD88, and IL-10 genes in intestinal tissues were also significantly increased (*p* < 0.05). Conversely, both the CYC and YP groups showed significantly reduced relative transcription levels of the IL-12, IL-6, IL-1β, and TNF-α genes in intestinal tissues (*p* < 0.05) (Figure 4). 

### 3.5. CYC and YPs Modulate CD4 and CD8 Expression in the Jejunum of Lambs

**Figure 5:**
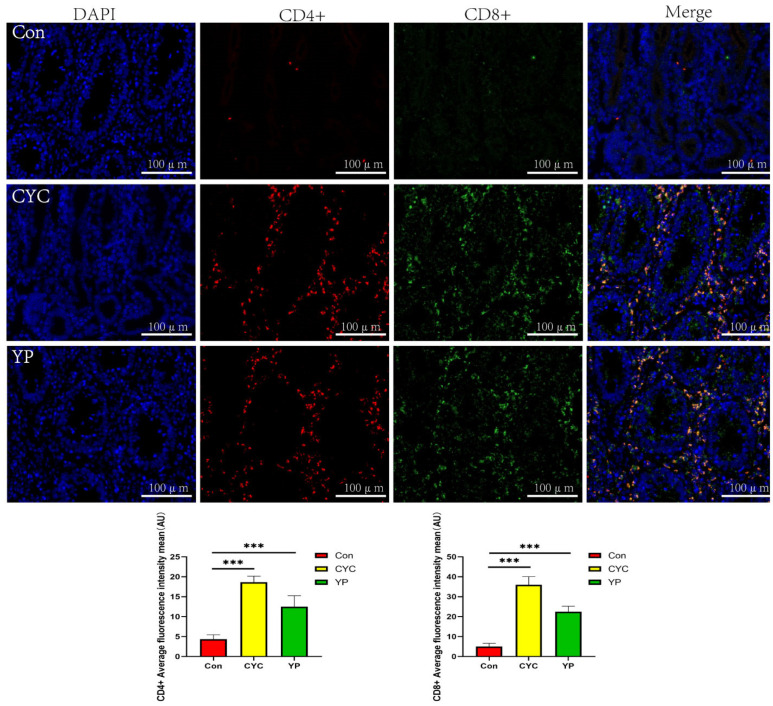
The effects of dietary CYC and YP supplementation on CD4 and CD8 expression in the intestinal tract of lambs. Con = basal diet; CYC = compound yeast culture; YP = yeast cell wall polysaccharide; *n* = 6/group; *** *p* < 0.001.

The numbers of CD4^+^ and CD8^+^ T lymphocytes in the intestinal tissues of lambs from each group were determined using immunofluorescence. Compared with the control group (Con group), the counts of CD4^+^ and CD8^+^ T cells in the intestinal tissues were significantly elevated in both the CYC group and the YP group (*p* < 0.001), with the CYC group exhibiting the highest numbers of these two T-cell subsets (Figure 5). These results indicate that both CYC and YPs can maintain intestinal immune homeostasis in lambs by modulating T-cell populations, and CYC shows the most pronounced effect.

### 3.6. CYC and YPs Modulate the Structure of the Intestinal Microbiota in Lambs

**Figure 6:**
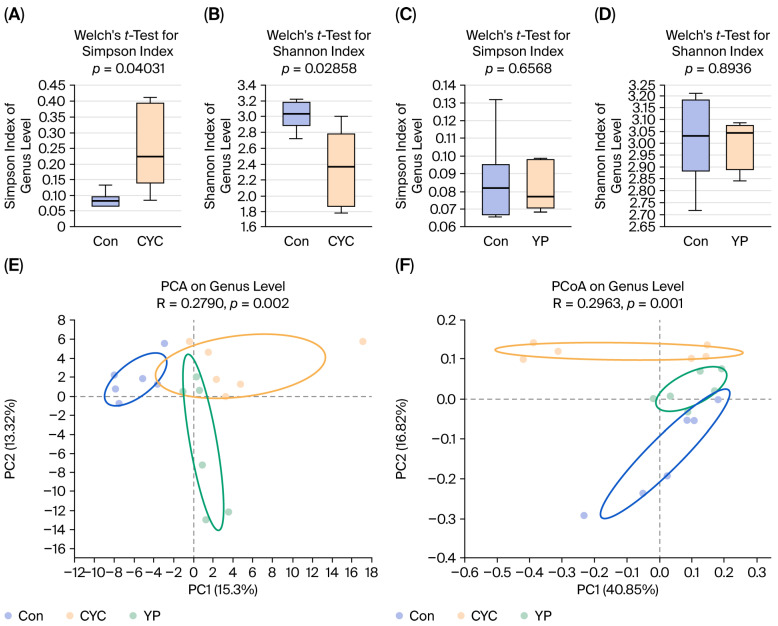
PCA and PCOA beta diversity plots in the Con, CYC, and YP groups. (**A**–**D**): Alpha diversity comparisons (Simpson and Shannon indices) between the Con vs. CYC and Con vs. YP groups using Welch’s *t*-test. (**E**): Principal component analysis (PCA) of genus-level bacterial community structure. (**F**): Principal coordinate analysis (PCoA) of genus-level bacterial community structure based on Bray–Curtis dissimilarity.

A 16S rRNA sequencing strategy was next utilized to clarify the effects of CYC and YP intake on the gut microbiome, achieving a coverage rate of >99.8% across the analyzed samples. The alpha diversity analysis showed that the Simpson index (*p* < 0.05) and Shannon index (*p* < 0.05) were significantly different between the Con and CYC groups (Figure 6A,B), while no significant differences were observed between the Con and YP groups (Figure 6C,D). In the YP group, markedly raised ACE and Chao1 diversity index values were noted relative to the Con and CYC groups (*p* < 0.05) (Table 6). The structure of the gut microbial community in these lambs was then examined through PCA and PcoA approaches (Figure 6E,F). At the species level, the first principal component (PC1) accounted for 15.3% of the overall sample variability with respect to detected microorganisms, while PC2 accounted for 13.32%. At the genus level, PC1 and PC2, respectively, accounted for 40.85% and 16.82% of the overall variability. These analyses highlighted clear trends toward sample clustering in the same group, although the spread of these samples was indicative of differences in the specific detected microbes among samples. Overall, these results suggested that dietary CYC or YP supplementation altered the makeup of the gut microbiome in lambs.

**Figure 7:**
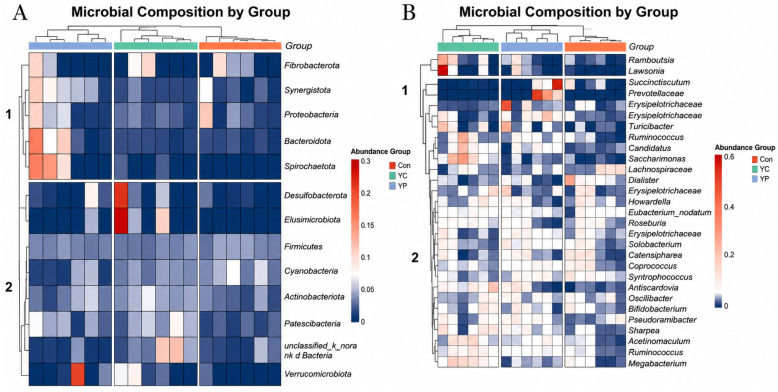
Analysis of the microbial composition of intestinal microbiota in lambs. (**A**) Microbial composition at the phylum level. (**B**) Microbial composition at the genus level.

**Figure 8:**
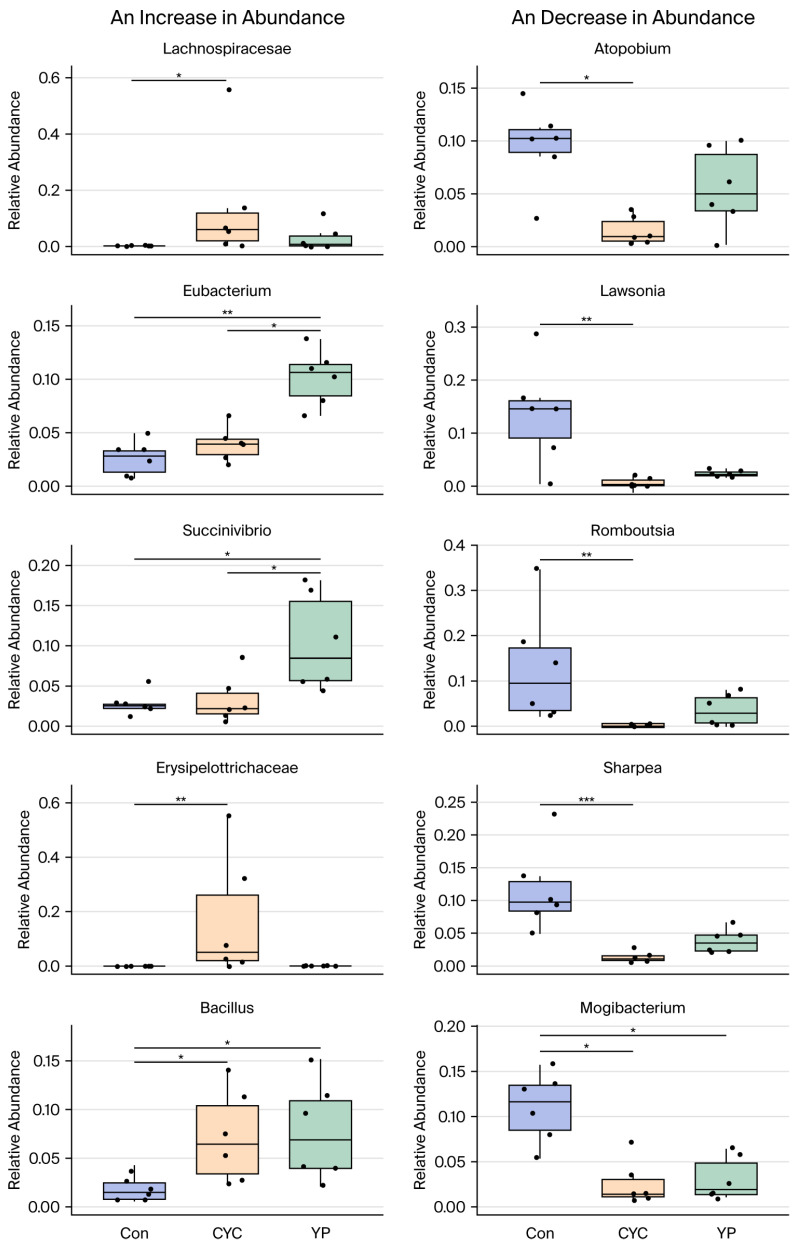
Analysis of species relative abundance at the genus level. Con = basal diet; CYC = compound yeast culture; YP = yeast cell wall polysaccharide; *n* = 6/group; * *p* < 0.05, ** *p* < 0.01, *** *p* < 0.001.

The thermal map of the intestinal flora of the lambs showed that the relative abundance of *Fibrobacterota* and *Elusimicrobia* showed an increasing trend (Figure 7A), whereas that of *Proteobacteria* and *Spirochaetota* showed a decreasing trend (Figure 7B). The relative abundances of *Lachnospiraceae* and *Erysipelotrichaceae* were significantly higher in the CYC group. The abundance of *Eubacterium* was markedly increased in the YP group (*p* < 0.01), and *Bacillus* was significantly elevated in both CYC and YP groups (*p* < 0.05). Conversely, the relative abundances of *Atopobium*, *Lawsonia*, *Romboutsia*, *Sharpea*, and *Mogibacterium* in the CYC group were significantly lower than in the Con group (*p* < 0.05; *p* < 0.01) (Figure 8). These changes suggest that CYC and YPs may modulate the composition of the intestinal microbiota in association with improved intestinal homeostasis.

### 3.7. Evaluation of the Correlations Between Intestinal Microbes, Immune Factors, and Tight Junction Proteins in the Jejunum

**Figure 9:**
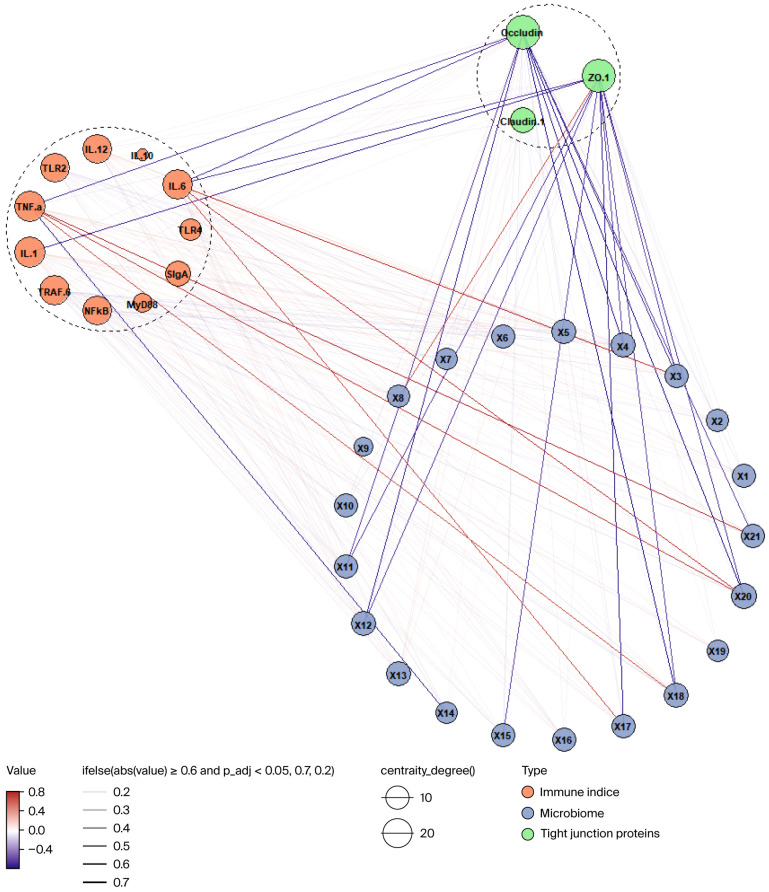
Analysis of the correlation between immune indicators, microbiota and tight junction proteins. X1 = *Actinobacteriota*; X2 = *Patescibacteria*; X3 = *Romboutsia*; X4 = *Marvinbryantia*; X5 = *Succiniclasticum*; X6 = *Saccharofermentans*; X7 = *Anaerovoracaceae*; X8 = *Pseudoscardovia*; X9 = *Paenibacillus*; X10 = *Enterorhabdus*; X11 = *Atopobium*; X12 = *Eubacterium*; X13 = *Desulfovibrio*; X14 = *Lachnospiraceae*; X15 = *Lawsonia*; X16 = *Butyricicoccaceae*; X17 = *Erysipelottrichaceae*; X18 = *Prevotellaceae*; X19 = *Succinivibrio*; X20 = *Mogibacterium*; X21 = *Bacillus*.

**Figure 10:**
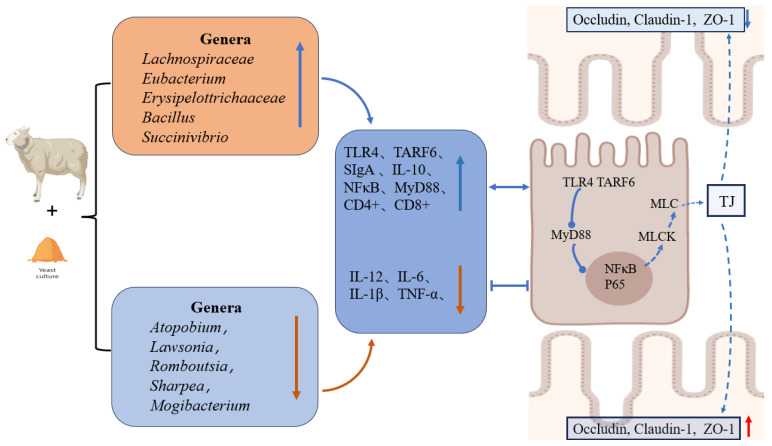
The proposed mechanisms through which supplemental CYC and YPs enhance intestinal barrier function in sheep. ↑ indicates upregulation/increase; ↓ indicates downregulation/decrease; blue arrows denote stimulatory effects; orange arrows denote inhibitory effects.

Spearman correlation analysis indicated that the abundance of *Lawsonia* was negatively correlated with ZO-1 expression (*r* = 0.604), and ZO-1 was negatively correlated with IL-1β (*r* = −0.788) (Figure 9). CYC increased the relative abundance of *Lachnospiraceae* (*p* < 0.05), which was negatively correlated with TNF-α expression (*r* = −0.581), while TNF-α was negatively correlated with occludin expression. Both CYC and YPs significantly reduced the abundance of *Mogibacterium*, which was positively correlated with TNF-α expression. CYC significantly decreased the relative abundance of *Romboutsia*, which was negatively correlated with occludin expression (*r* = −0.625). These results indicate that CYC and YPs may enhance intestinal barrier function by modulating microbial taxa that are associated with tight junction protein expression and inflammatory status. Moreover, both CYC and YPs can significantly reduce the abundance of *Mogibacterium* in the intestinal microbiota (*p* < 0.05), which is positively correlated with TNF-α expression (*r* = 0.482), and the latter is negatively correlated with that of occludin (*r* = −0.756). CYC significantly decreased the relative abundance of *Romboutsia* (*p* < 0.01), which is negatively correlated with the expression of occludin (*r* = −0.583), and occludin expression is negatively correlated with the expression of IL-6 (*r* = −0.622). In conclusion, the supplementation of CYC or YPs can modulate the intestinal flora, immune factors in the intestinal tissue, and intestinal tight junction proteins to maintain intestinal health by improving the intestinal barrier through mutual regulation and synergistic effects (Figure 10).

As the core active components of both CYC and YPs, β-glucan and mannan oligosaccharides share similar regulatory mechanisms. This diagram uses CYC as a representative to illustrate the key signaling pathways and interactions involved in improving the intestinal physical barrier, immune barrier, and biological barrier function.TJ = Tight junction protein; CYC = compound yeast culture; YP = yeast cell wall polysaccharide.

## 4. Discussion

In this study, CYC and YP supplementation significantly increased the intestinal expression of tight junction proteins including occludin, claudin-1, and ZO-1 in lambs compared to the control group. These findings align with Ren Shunan et al.’s report that MLCK regulates ZO-1, mediates claudin-1, and activates occludin to modulate tight junction structure [23]. In addition, downregulation of IL-1β and TNF-α increases permeability by inducing the redistribution of MLCK and tight junction proteins [24]. The changing trends in immune factors and tight junction proteins and the correlation between them we observed in this experiment are consistent with the above discoveries. In this study, the transcription levels of the IL-1β, IL-6, and TNF-α genes decreased in the intestinal tissues of lambs after supplementation with CYC, and the expression levels of occludin, claudin-1, and ZO-1 increased. These results suggest that CYC may enhance the levels of intestinal tight junction proteins in lambs by regulating inflammatory factors to a certain extent. In addition to the expression of such cytokines, we also detected the effects of the dietary addition of CYC and YPs on the immune activity of T cells. Th17 cells, a newly discovered subset of CD4^+^ cells, promote the secretion of IL-6, while IFN-γ, IL-4, and IL-2 can inhibit the differentiation of Th17 [25]. Within a certain range, the increase in the CD4^+^ cell percentage indicates that the animal’s immune function is better. In this study, the immunofluorescence method was used to observe that the counts of CD4^+^ and CD8^+^ T cells in the intestinal tissues were significantly elevated in both the CYC group and the YP group, with the CYC group exhibiting the highest numbers of these two T-cell subsets, and we speculated that CYC might affect the function of intestinal T cells in lambs. Intestinal tight junction protein expression and intestinal villus development were better; thus, the digestive and absorption capacity was increased, intestinal inflammatory factors were decreased, and the intestinal mucosal immune index SIgA expression was high, all of which, to a large extent, reduced the risk of intestinal inflammation and oxidative stress in lambs.

According to extensive literature, *Firmicutes* and *Bacteroidetes* can account for more than 80% of the microbiota in the intestinal tract of animals. In enteritis disease, the abundance of *Bacteroidetes* has been shown to decrease significantly, while *Proteobacteria* increased, leading to intestinal damage and the aggravation of inflammation [26]. Therefore, *Proteobacteria* can be used as microbial signal indicators of disease. In addition, the oral administration of *Prevotellaceae* can enhance the intestinal health of calves [27]. In this study, dietary supplementation with CYC or YPs modified the structure of the lamb intestinal microbiota. The relative abundance of *Prevotellaceae* and Bacillus was increased, while that of *Proteobacteria* and *Spirochaetota* was decreased. It should be emphasized that *Proteobacteria* are not uniformly associated with disadvantageous effects, and their roles are context-dependent. However, the observed reduction in *Proteobacteria* in this study coincided with lower inflammatory cytokine levels, suggesting a relationship with intestinal homeostasis. Some researchers demonstrated that compound yeast culture exhibits a more pronounced effect than *S. cerevisiae* in elevating the immunoglobulin levels and further elucidated the positive impact of compound yeast culture and *S. cerevisiae* on maintaining the equilibrium of the intestinal flora in lambs [28]. Moreover, their study verified that compound yeast culture can augment the quantity of beneficial bacteria (such as *Lactobacillus* and *Bifidobacterium*) in the rectum of lambs, while concurrently reducing the content of harmful bacteria (such as *E. coli* and *Salmonella*). This above outcome is in accordance with the discoveries of our research. The potential mechanism is as follows: after colonization, *Bacillus* and *Prevotellaceae* can rapidly consume the oxygen in the gastrointestinal tract, thereby creating a hypoxic microenvironment. This hypoxic state is conducive to promoting the colonization of beneficial anaerobic bacteria (such as *Lactobacillus*), and through the generation of organic acids, the pH of the gastrointestinal tract was lowered, thereby inhibiting the growth of pathogenic bacteria [29]. Studies have demonstrated that an elevation in the abundance of *Lawsonia* within the intestine can disrupt the β-catenin/Wnt and Notch signaling pathways in intestinal stem cells, thereby eliciting the abnormal proliferation of intestinal cells [30]. This alteration further gives rise to thickening of the ileal wall and diminished secretion of Muc2 mucin, and ultimately undermines the intestinal barrier function, facilitating the invasion of pathogens into the intestinal tissue [31]. The results of this study reveal that CYC supplementation significantly decreased the abundance of *Lawsonia*, which was negatively correlated with ZO-1 expression. *Lachnospiraceae* was enriched in the CYC group and negatively correlated with TNF-α, consistent with a context-dependent role in supporting intestinal barrier function. These findings reveal coordinated interactions among intestinal microbiota, tight junction proteins, and immune factors, rather than simplistic beneficial or harmful effects of individual taxa.

Studies have shown that YPs have great potential as an antioxidant and immunomodulator in food, and the regulatory effects of oral beta-glucan on the immune system, intestinal flora, and metabolic diseases have been investigated [32]. Our team elucidated that yeast β-glucan can inhibit LPS-induced oxidative damage to lamb lymphocytes, and explored the immune mechanism of β-glucan via the Nrf2/HO-1 signaling pathway based on mouse macrophages [33]. One study showed that dietary supplementation with mannan reduced the passive transfer of G protein, enhanced antioxidant capacity and immunity, and improved the intestinal flora of neonatal goats [34]. In a study concerning the stimulation of peripheral blood mononuclear cells (PBMCs) in lambs by lipopolysaccharide (LPS) and lipoteichoic acid (LTA), it has been unequivocally established that TLR2, TLR4, and the NFκB pathway play a crucial role in the early immune response, potentially transmitting signals through downstream molecules such as MyD88 and TRAF6 [35]. This study revealed that the TLR2 expression level was significantly enhanced in the YP group, while the expression levels of TLR4 and NFκB in the CYC group were significantly higher than those in the Con group. Based on the aforementioned results, we hypothesize that CYC and YPs may act on the intestinal immune barrier by stimulating TLR2 and TLR4/NFκB/MyD88-related signal transduction. In view of the above findings, β-glucan and mannan, as the main components of CYC and YPs in this study, may be responsible for the increased production performance, changes in intestinal immune factors, increased expression of tight junction proteins, and changes in microflora structure. Because CYC or YPs are digested by the ruminant’s forestomach, it is unknown which components are decomposed and reach the intestine. At present, there are few studies on the specific mechanisms by which CYC and YPs act in the intestine of ruminants. Therefore, our team has constructed immortalized small intestinal epithelial cells from lambs. A follow-up study should investigate the mechanism by which CYC and YPs act on the physical, immune, biological, and chemical barriers of the intestine.

## 5. Conclusions

The dietary addition of CYC may promote the development of intestinal villi and improve the expression of tight junction proteins such as occludin, claudin-1, and ZO-1 in the intestinal tissue of lambs. The transcriptional levels of the TLR4, SigA, and MyD88 genes in the jejunal tissue in the CYC group were increased, the IL-12 and IL-1β genes were decreased, and the levels of CD4 and CD8 in the CYC group were increased, indicating that CYC regulated the gene abundance of inflammatory factors in intestinal tissue and the secretion function of T cells to a certain extent. After 30 days of CYC supplementation, the relative abundances of *Lachnospiraceae*, *Eubacterium*, *Prevotellaceae*, *Erysipelotrichaceae*, and *Bacillus* in the intestinal tract of lambs in the CYC group were increased compared with the control group, while those of *Atopobium*, *Lawsonia*, *Romboutsia*, *sharpea*, and *Mogibaaterium* were decreased, indicating that CYC can change the intestinal flora structure of lambs. All in all, the supplementation of the diet with CYC can facilitate the development of small intestinal villi in lambs and might regulate the immune barrier function through ameliorating the structure of the intestinal flora and promoting the expression of tight junction proteins. These data can provide a theoretical basis for the application of CYC in Mongolian ram lambs.

## Figures and Tables

**Table 1:** Compound yeast culture and yeast cell wall polysaccharide nutrient composition.

Items	CYC	YPs
Crude protein	20.39	31
Dry matter, %	92.58	95.3
Neutral detergent fiber, %	33.45	73
Acid detergent fiber, %	19.2	52
β-Glucan, %	10.5	42
Mannan, %	9	20
Live yeast cells, CFU/g	6.8 × 10^4^	-

CYC = compound yeast culture; YPs = yeast cell wall polysaccharides.

**Table 2:** Composition and dietary nutrient composition (%, DM basis).

Items	Groups		
	Con	CYC	YP
Ingredients			
Peanut vine	23	23	23
Cornstalk	5	5	5
Sunflower seed skin	4	4	4
Alfalfa meal	10	10	10
Corn	27	27	27
Soybean meal	10	10	10
Germ meal	5.5	5.5	5.5
Cotton meal	5	5	5
Wheat bran	2	2	2
Peanut cake	4	4	4
NaCl	0.5	0.5	0.5
NaHCO_3_	0.5	0.5	0.5
CaHPO_4_	0.5	0.5	0.5
4% premix ^1^	3	3	3
Total	100	100	100
Nutrient levels (%, DM basis)			
Metabolizable energy, MJ/kg ^2^	10.48	11.00	10.36
Dry matter ^3^	88.5	89.77	88.21
Crude protein ^4^	15.81	15.91	15.29
Organic matter ^5^	95.6	96.89	95.23
Neutral detergent fiber	32.68	32.86	32.56
Acid detergent fiber	21.76	22.25	21.11
Ca	2	2	2
P	1	1	1

^1^ The premix contained the following (per kg): Fe 60 mg, Cu 12 mg, Zn 60 mg, Mn 45 mg, nicotinic acid 60 mg, I 0.6 mg, Se 0.2 mg, VA 3500 IU, VD 1200 IU, VE 20 IU, Ca 2 g, P 1 g, Co 20 mg, and NaCl 5 g. ^2^ Metabolizable energy was calculated as described (NRC, 2001), and the others were measured values. ^3^ Dry matter: Determined by oven-drying at 105 °C (AOAC method 930.15, 2000). ^4^ Crude protein: Determined by the micro-Kjeldahl method (K1100, Hanon instruments, Shandong, China) (AOAC method 976.05, 2000). ^5^ Organic matter: Determined by the muffle furnace constant-temperature calcination method (AOAC method 942.05, 2005). Con = basal diet; CYC = compound yeast culture; YP = yeast cell wall polysaccharides.

**Table 3:** Primer sequences.

Gene	Sequence (5′-3′)	GenBank No.
V3-V4	341F: 5′-CCTACGGGNGGCWGCAG-3′	Reference
	805R: (5′-GACTACHVGGGTATCTAATCC-3′)	
MyD88	F: ATGGTGGTGGTTGTCTCTGAC	GQ221044.1
	R: GGAACTCTTTCTTCATTGGCTTGT	
TLR-2	F: CAAGAGGAAGCCCAGGAAG	DQ890157.1
	R: TGGACCATGAGGTTCTCCA	
TLR-4	F: TGCTGGCTGCAAAAAGTATG	HQ343416.1
	R: CCCTGTAGTGAAGGCAGAGC	
TRAF-6	F: TCAGAGAACAGATGCCCAAT	XM_012134166.2
	R: GCGTGCCAAGTGATTCCT	
IL-10	F: AGGAAAAAGATGGATGCTTCCA	NM_001009392
	R: GACCAGCAGTGGTTTTGATCAA	
IL-12	F: CGTCTTCCTGGGACGTTTTAG	NM_001009465
	R: CTGCGTATGGCTTCTTTAGGG	
NF-κB	F: ATACGTCGGCCGTGTCTAT	XM_005226864.2
	R: GGAACTGTGATCCGTGTAG	
TNF-α	F: ACACCATGAGCACCAAAAGC	NM_001024860.1
	R: AGGCACAAGCAACTTCTGGA	
IL-1β	F: GGCAGATGATAATTGGCGTTAC	NW_025421343.1
	R: CTCGCGGGGCAGCTCTTGA	
IL-6	F: AGGAAAAAGATGGATGCTTCCA	NM_001009392
	R: GACCAGCAGTGGTTTTGATCAA	
sIgA	F: GGCAGATGATAATTGGCGTTAC	AF024645.1
	R: CTCGCGGGGCAGCTCTTGA	
β-actin	F: GCTCTTCCAGCCGTCCTT	NM_001009784.1
	R: TGAAGGTGGTCTCGTGAATGC	

MyD88 = myeloid differentiation primary response gene 88; TLR2 = Toll-like receptor 2; TLR4 = Toll-like receptor 4; TRAF6 = recombinant TNF receptor associated factor 6; IL-10 = interleukin-10; IL-12 = interleukin-12; NF-κB = nuclear factor kappa-B; TNF-α = tumor necrosis factor-α; IL-1β = interleukin-1β; IL-6 = interleukin-6; SigA secretory immunoglobulin A.

**Table 4:** The effects of CYC and YPs on growth performance of lambs (kg).

Item	Con	CYC	YPs	*p*-Value
Initial weight	19.28 ± 2.82	18.39 ± 5.55	18.28 ± 1.64	0.48
Initial intake	0.91 ± 0.22	0.89 ± 0.15	0.88 ± 0.35	0.183
30 d weight	24.83 ± 4.52	26.17 ± 5.69	25.89 ± 3.01	0.178
30 d intake	1.14 ± 0.26 ^b^	1.19 ± 0.34 ^a^	1.18 ± 0.36 ^a^	0.045
ADG (0~30 d)	0.19 ± 0.03 ^c^	0.26 ± 0.06 ^a^	0.25 ± 0.05 ^b^	0.033
ADFI (0~30 d)	1.03 ± 0.27	1.04 ± 0.31	1.03 ± 0.22	0.072
F/G	5.42 ± 0.22 ^a^	4.00 ± 0.38 ^b^	4.12 ± 0.24 ^b^	0.041

ADG = average daily gain; ADFI = average daily feed intake; F/G = the ratio of feed intake to body weight gain. ^a–c^ Different letters in a given row indicate significant differences at *p* < 0.05. Con = basal diet; CYC = compound yeast culture; YPs = yeast cell wall polysaccharides.

**Table 5:** The impact of CYC and YPs on lambs’ intestinal morphology.

	Con	CYC	YPs	*p*-Value
Villous height (μ)				
Duodenum	523.54 ± 15.03 ^b^	552.50 ± 12.36 ^a^	532.32 ± 16.12 ^b^	0.043
Jejunum	516.26 ± 23.11	532.21 ± 13.41	525.25 ± 25.34	0.178
Ileum	407.38 ± 19.23	425.44 ± 11.40	395.54 ± 21.32	0.064
Crypt depth (μm)				
Duodenum	474.36 ± 11.33 ^b^	486.71 ± 13.33 ^a^	481.86 ± 18.05 ^a^	0.029
Jejunum	386.25 ± 21.22	365.53 ± 15.28	388.30 ± 18.00	0.097
Ileum	327.42 ± 30.32 ^b^	346.21 ± 23.21 ^b^	371.23 ± 26.12 ^a^	0.025
VCR				
Duodenum	1.10 ± 0.07	1.14 ± 0.15	1.11 ± 0.11	0.214
Jejunum	1.33 ± 0.12 ^b^	1.46 ± 0.31 ^a^	1.35 ± 0.18 ^b^	0.048
Ileum	1.24 ± 0.23 ^b^	1.23 ± 0.09 ^a^	1.06 ± 0.05 ^c^	0.019

VCR = Villous height to crypt depth ratio. ^a–c^ Different letters in a given row indicate significant differences at *p* < 0.05. Con = basal diet; CYC = compound yeast culture; YPs = yeast cell wall polysaccharides. *n* = 6/group.

**Table 6:** The impact of dietary CYC and YP supplementation on alpha diversity indices for the gut microbiota in lambs.

Estimators/Sample	Con	CYC	YPs	*p*-Value
Shannon	3.39 ± 0.2 ^a^	2.57 ± 0.18 ^b^	3.38 ± 0.15 ^a^	0.002
Simpson	0.06 ± 0.02 ^b^	0.23 ± 0.01 ^a^	0.06 ± 0.02 ^b^	0.003
Ace	229.99 ± 11.23 ^b^	218.9 ± 8.65 ^b^	241.44 ± 7.95 ^a^	0.03
Chao1	201.78 ± 22.64 ^b^	201.74 ± 18.97 ^b^	233.57 ± 15.51 ^a^	0.006

^a,b^ Different letters in a given row indicate significant differences at *p* < 0.05. Con = basal diet; CYC = compound yeast culture; YPs = yeast cell wall polysaccharides.

## Data Availability

The data that support the findings of this study are available from the corresponding author upon reasonable request.
